# Cancer

**Published:** 1906-02-03

**Authors:** 


### CANCER.

(*Continued from page* 291.)

**Criticism** of the work of the Imperial Cancer Research Fund and Bashford's scientific Report is supplied by Moore and Walker,[6] whose investigations with Farmer on heterotype mitosis have been confirmed by this very authority. In the first place, they allege that the views they have expressed are misrepresented in Bashford's Report, mainly in the direction of amplification. These writers merely state that the tissues of malignant growths present, on the supervention of malignancy, some attributes of reproductive tissues not that the origin of malignant growths is by transformation of tissue cells into modified reproductive cells. They also deny that they ascribe proliferative and other powers to "modified reproductive or gametoid" tissue. In fact, they do not consider that these properties of malignant growths are the necessary consequence of the appearance of gametoid tissue at abnormal sites. Moreover, they do not consider "reduced tissues"—namely, tissue produced by heterotype mitosis—as constituting the essential part of malignant growths. Various other points are dealt with by the writers, but the most important difference between themselves and Bashford seems to lie in the fact that, whereas the latter observer reports that after heterotype mitosis he has observed nuclear fusion, they have observed no such process, and are not convinced of its occurrence by the evidence he brings forward in support of it. They are, however, inclined to credit certain changes in the leucocytes with the effects he attributes to the nuclear fusions he describes. Further, they deny that continuous growth is a special characteristic of cancer cells, and that infiltration and destruction are a mere consequence of the same. They also protest against the statement of Bashford that the attributes of cancer are consequences of its powers of unlimited growth. The view of the importance of the leucocyte in the causation of malignancy is also supported by Forbes Ross,[7] who, working independently, was led to somewhat similar conclusions. Briefly, his theory is that cancerous growth is due to alterations in cell polarity consequent on the withdrawal of nervous influence and the stimulation of certain leucocytes. Cell polarity is of two varieties—the morphological, based on an axis drawn through the nucleus and centrosome, with the former nearest the attached end; and the physiological, based on an axis drawn through the epithelial cell from its basal end (the source of its nervous and food supply) and its free end, by which it discharges its functions. These axes correspond, and are at right angles to the surface on which the cell grows. The polarity may be permanently fixed, as in ciliated cells, goblet cells, etc., and these cells are never the starting-point of cancerous growth, though they may be included in a cancerous tumour. In that case they retard the development of malignancy. Of epithelial cells, those whose nerve supply is in the form of end bulbs at the side of the cells, are alone the starting-point of cancer; while of mesoblastic cells, those whose polarity is permanently fixed—such as striped muscle cells—are not able to assume cancerous properties, while those such as connective tissue corpuscles, whose polarity is various or lost, are able to do so. Fixed polarity and high specialisation as in mesoblastic tissue are thus antagonistic to the assumption of malignancy. In this respect hypoblastic and epiblastic cells which, if damaged, can be regenerated, are comparable to mesoblastic spindle cells, the result of damage to differentiated mesoblast. The exception to this rule is the epiblastic nerve cell. Thus mesoblastic tissues are of fixed and permanent polarity, and only generate cancer in their undeveloped condition—namely, when sarcoma arises from undifferentiated cells. Forbes Ross considers that while some leucocytes aid in the cure of cancers, others (possibly the polynuclears) are the active agents in producing cancer by disturbing the polarity of epithelial cells. This altered polarity, as shown by the abnormal relation of the centrosome and nucleus, he has observed in epitheliomata and other cancers associated with the presence of numerous leucocytes. He thinks it possible that when normal tissues are damaged, and the *débris* is carried away by leucocytes, the polynuclears have the property of stimulating growth (by altering the polarity of the other cells) and the mononuclears of restraining it; and this he believes is supported by histological evidence.

[6] Lancet, July 22, 1905, p. 246. [7] Brit. Med. Jour., Oct. 28 1905, p. 1101.

